# MALDI-TOF MS typing enables the classification of brewing yeasts of the genus *Saccharomyces* to major beer styles

**DOI:** 10.1371/journal.pone.0181694

**Published:** 2017-08-09

**Authors:** Alexander Lauterbach, Julia C. Usbeck, Jürgen Behr, Rudi F. Vogel

**Affiliations:** 1 Lehrstuhl für Technische Mikrobiologie, Technische Universität München, Freising, Germany; 2 Bavarian Center for Biomolecular Mass Spectrometry, Freising, Germany; University of Leicester, UNITED KINGDOM

## Abstract

Brewing yeasts of the genus *Saccharomyces* are either available from yeast distributor centers or from breweries employing their own “in-house strains”. During the last years, the classification and characterization of yeasts of the genus *Saccharomyces* was achieved by using biochemical and DNA-based methods. The current lack of fast, cost-effective and simple methods to classify brewing yeasts to a beer type, may be closed by Matrix Assisted Laser Desorption/Ionization–Time-Of-Flight Mass Spectrometry (MALDI-TOF MS) upon establishment of a database based on sub-proteome spectra from reference strains of brewing yeasts. In this study an extendable “brewing yeast” spectra database was established including 52 brewing yeast strains of the most important types of bottom- and top-fermenting strains as well as beer-spoiling *S*. *cerevisiae* var. *diastaticus* strains. 1560 single spectra, prepared with a standardized sample preparation method, were finally compared against the established database and investigated by bioinformatic analyses for similarities and distinctions. A 100% separation between bottom-, top-fermenting and *S*. *cerevisiae* var. *diastaticus* strains was achieved. Differentiation between Alt and Kölsch strains was not achieved because of the high similarity of their protein patterns. Whereas the Ale strains show a high degree of dissimilarity with regard to their sub-proteome. These results were supported by MDS and DAPC analysis of all recorded spectra. Within five clusters of beer types that were distinguished, and the wheat beer (WB) cluster has a clear separation from other groups. With the establishment of this MALDI-TOF MS spectra database proof of concept is provided of the discriminatory power of this technique to classify brewing yeasts into different major beer types in a rapid, easy way, and focus brewing trails accordingly. It can be extended to yeasts for specialty beer types and other applications including wine making or baking.

## Introduction

The major parameters defining a beer type comprise process parameters and the ingredients malt, hops and yeast used [1]. In many countries further parameters can be varied including the use of unmalted grains, enzymes and other additives [2]. The process of fermentation initiation by a selected strain was unknown at that time and mostly a wild fermentation occurred. Along with the discovery of the fermentation of sugars by yeasts between 1789 to 1839 and the development of pure yeasts for a monitored brewing, the purity law was expanded within the beer taxes law [3–5]. Since 1906, it has been fixed that in Germany brewers are only allowed to use malt, hops, water and yeasts (according to the German brewer association). While the variation of malts has a long tradition and the exploitation of new hop varieties for craft beer brewing is upcoming in recent years, most breweries only use one single or a very small number of brewing yeasts. While exploitation of the yeast variety is of importance for every brewer, it is invaluable for German brewers and others, who want to stick with the original beer ingredients.

Generally, brewing yeasts are divided to top-fermenting *Saccharomyces* (*S*.) *cerevisiae* and bottom-fermenting *S*. *pastorianus* strains [2]. Annemüller, Manger [3] explained that properties such as growth and fermentation temperature, forming of fermentation by-products or cell complexes are the main differences of these two species. Nevertheless, both species belong to the *Saccharomyces* genus, which contains nine *Saccharomyces* species including hybrids: *S*. *eubayanus*, *S*. *arboricola*, *S*. *bayanus*, *S*. *cariocanus*, *S*. *cerevisiae*, *S*. *kudriavzevii*, *S*. *mikatae*, *S*. *paradoxus and S*. *pastorianus* [6–8]. According to Libkind, Hittinger [6], Peris, Sylvester [9] and Bing, Han [10] the hybridization of *S*. *cerevisiae* and *S*. *eubayanus* as well as the adjustment to the brewing environment has resulted in the actual complex of *S*. *pastorianus* hybrids. Strains of these hybrids can be differentiated along specific properties, namely their flocculent behavior [11]. The flocculent behavior has a desirable meaning for brewers, since flocculation leads to a bright beer, and a low degree of attenuation, whereas the use of powdery strains results in a higher degree of attenuation instead [12, 13]. The flocculent behavior plays an important role for the production of lager beer as the brewers have to choose between flocculent and powdery bottom-fermenting yeast strains and the resulting fermentation behavior. Whereas top-fermenting *S*. *cerevisiae* strains, used in the brewery, have usually a powdery behavior [13] and are differentiated to the creation of a wide variety of aromatic compounds [14–16].

Brewing yeasts are mainly classified in top- and bottom-fermenting yeast. Especially top-fermenting yeasts are grouped by yeast centers in beer types such as wheat beer, ale/stout, German “Altbier” or “Kölsch”. Brewers have the opportunity to buy various yeast strains, nevertheless there are some who isolated their in-house brewing yeast by themselves. The characterization of such in-house strains is mostly based on morphological, physiological and fermentation properties. Unfortunately, there is no information on the genomic or proteomic background and the beer type to which the yeast strain belongs.

During the last years, the classification and characterization of yeasts of the genus *Saccharomyces* was achieved along with biochemical and mostly DNA-based methods. Some experiments included the karyotyping of chromosomes by pulsed filed gel electrophoresis to describe brewing yeast strains, new lager yeast strains or hybrids [17–20]. Amplified fragment-length polymorphism (AFLP) was used to investigate genetic variation of *Saccharomyces* and non-*Saccharomyces* yeasts [21, 22]. Experiments based on random amplified polymorphic DNA (RAPD) could differentiate strains within the species *Saccharomyces cerevisiae* [23] or distinguish top-fermenting variants from other yeasts [24]. Timmins, Quain [25] have shown the discrimination of ale and lager yeasts by pyrolysis mass spectrometry and Fourier transform infrared spectroscopy. The genetic diversity of *S*. *cerevisiae* strains from different origins like beer, wine and bread based on genomics were presented by Legras, Merdinoglu [26], Gonçalves, Pontes [27] and Gallone, Steensels [28].

However, the selection process and classification of brewing yeasts to a specific beer type is elaborate and based on trial and error. Currently there is a lack of fast, cost-effective and simple methods to assign brewing yeasts to a beer type. Matrix Assisted Laser Desorption/Ionization–Time-Of-Flight Mass Spectrometry (MALDI-TOF MS) may close this gap upon establishment of a database based on brewing yeasts if the discriminatory power allows respective sorting of the strains. Besides the simple identification of bacteria on species or for some species even on strain level [29–31], MALDI-TOF MS has been used for the classification of yeasts from various genera from different environments like clinical or food-borne samples [32–34]. Former studies showed that MALDI-TOF MS has also qualified to characterize yeast strains of *Saccharomyces* along their sub-proteome spectra showing their relationships, application potential and physiological status [35–37]. MALDI-TOF MS and specifically amplified polymorphic DNA (SAPD-PCR) was used to differentiate species of the genus *Saccharomyces* [35]. Usbeck, Wilde [36] demonstrated the possibility of a wine yeast typing of *Saccharomyces* strains and Moothoo-Padayachie, Kandappa [37] visualized the variety within the sub-proteome spectra of *Saccharomyces cerevisiae* strains from different industrial parts by MALDI-TOF MS.

The aim of this study was to provide proof of concept for the discriminatory power of MALDI-TOF MS to categorize *Saccharomyces* yeasts along their potential for specific applications. Therefore, a brewing yeast database should be established with spectra obtained under standardized conditions by MALDI-TOF MS, which includes 52 commercial *Saccharomyces* yeast strains obtained from a yeast supplier and currently used in the brewing sector for the production of the major beer types worldwide. The database contains not only well characterized top- and bottom-fermenting yeast strains but also strains of the beer spoilage variety *S*. *cerevisiae* var. *diastaticus* [38]. Bioinformatics analyses of the sub-proteome spectra should provide information about their relationships, differences and similarities and possible assignment to a beer type of the *Saccharomyces* strains within the recorded spectra. This should enable brewers to match their in-house brewing yeast to a beer type by a fast, reliable and low running cost method, and focus brewing trails on most promising strains. Upon proof of concept in this communication, this database could also be extended to further yeast strains of the genus *Saccharomyces* comprising strains of specialty beers, winery, distillery or bakery.

## Materials and methods

### Strains

32 top-fermenting brewing yeast strains of the species *Saccharomyces* (*S*.) *cerevisiae*, 7 yeast strains of *S*. *cerevisiae* var. *diastaticus* and 13 bottom-fermenting *S*. *pastorianus* strains were obtained by the Research Center Weihenstephan for Brewing and Food quality (BLQ) (Table 1).

**Table 1:** Brewing yeast obtained from the BLQ. *TMW (= Technische Mikrobiologie Weihenstephan); TUM (Technische Universität München); the column one represents the species identification of the yeast supplier as well as by MALDI-TOF MS identification; the column four and five represent the beer types*: *WB = wheat beer*, *Alt = Alt-beer*, *Dias = Saccharomyces cerevisiae* var. *diastaticus; the last column explains the origin of each yeast strain (if available)*.

Species	TMW	TUM (BLQ)	Brewer experience	Classification by MALDI	Origin / Isolation
*Saccharomyces cerevisiae*	3.0250	68	WB	WB	Freising-Weihenstephan, Germany
*Saccharomyces cerevisiae*	3.0251	127	WB	WB	Freising-Weihenstephan, Germany
*Saccharomyces cerevisiae*	3.0252	148	Alt	Alt	Dusseldorf, Germany
*Saccharomyces cerevisiae*	3.0253	149	WB	WB	Munich, Germany
*Saccharomyces cerevisiae*	3.0254	165	Kölsch	Ale	Burton‐upon‐Trend, Great Britain
*Saccharomyces cerevisiae*	3.0255	175	WB	WB	Freising-Weihenstephan, Germany
*Saccharomyces cerevisiae*	3.0256	177	Kölsch	Kölsch	Krefeld, Germany
*Saccharomyces cerevisiae*	3.0257	184	Alt	Alt	Dusseldorf, Germany
*Saccharomyces cerevisiae*	3.0258	205	WB	WB	Würzburg, Germany
*Saccharomyces cerevisiae*	3.0259	308	Alt	Alt	Rhineland‐Palatinate, Germany
*Saccharomyces cerevisiae*	3.0260	210	Ale	Ale	Great Britain
*Saccharomyces cerevisiae*	3.0261	211	Ale	Ale	Great Britain
*Saccharomyces cerevisiae*	3.0262	213	Ale	Ale	Great Britain
*Saccharomyces cerevisiae*	3.0332a	998	Kölsch	Alt	Cologne, Germany
*Saccharomyces cerevisiae*	3.0332n	552	Kölsch	Alt	Cologne, Germany
*Saccharomyces cerevisiae*	3.0336	192	Alt	Alt	Dusseldorf, Germany
*Saccharomyces cerevisiae*	3.0337	338	Alt	Alt	Dusseldorf, Germany
*Saccharomyces cerevisiae*	3.0338	503	Ale	Ale	unknown, USA
*Saccharomyces cerevisiae*	3.0339	506	Ale	Ale	Great Britain
*Saccharomyces cerevisiae*	3.0343	505	WB	WB	Bavaria, Germany
*Saccharomyces cerevisiae*	3.0634	341	Alt	Alt	North Rhine-Westphalia, Germany
*Saccharomyces cerevisiae*	3.0635	431	Alt	Kölsch	North Rhine-Westphalia, Germany
*Saccharomyces cerevisiae*	3.0636	508	Ale	Ale	Ireland
*Saccharomyces cerevisiae*	3.0637	510	Ale	Ale	Great Britain
*Saccharomyces cerevisiae*	3.0666	220	WB	WB	Bavaria, Germany
*Saccharomyces cerevisiae*	3.0667	214	WB	WB	Bavaria, Germany
*Saccharomyces cerevisiae*	3.0668	513	Ale	Alt	unknown, USA
*Saccharomyces cerevisiae*	3.0669	454	WB	WB	Bavaria, Germany
*Saccharomyces cerevisiae*	3.0672	478	Ale	Ale	unknown, USA
*Saccharomyces cerevisiae*	3.0674	457	WB	WB	Bavaria, Germany
*Saccharomyces cerevisiae*	3.0675	174	Alt	Alt	Mühlheim, Germany
*Saccharomyces cerevisiae*	-	FK28	Kölsch	Alt	North Rhine-Westphalia, Germany
*Saccharomyces cerevisiae* var. *diastaticus*	3.0273	3-D-2	Dias	Dias	Northern Germany, Germany
*Saccharomyces cerevisiae* var. *diastaticus*	3.0274	3-H-2	Dias	Dias	Northern Germany, Germany
*Saccharomyces cerevisiae* var. *diastaticus*	3.0624	PI BB 105	Dias	Dias	unknown
*Saccharomyces cerevisiae* var. *diastaticus*	3.0625	71	Dias	Dias	North Rhine-Westphalia, Germany
*Saccharomyces cerevisiae* var. *diastaticus*	3.0628	DSMZ 70487	Dias	Dias	super-attenuated beer
*Saccharomyces cerevisiae* var. *diastaticus*	3.0811	PI BB 121	Dias	Dias	unknown
*Saccharomyces cerevisiae* var. *diastaticus*	3.0812	1-H-7	Dias	Dias	Bavaria, Germany
*Saccharomyces pastorianus*	3.0275	34/70	Lager	Lager	Freising-Weihenstephan, Germany
*Saccharomyces pastorianus*	3.0276	34/78	Lager	Lager	Freising-Weihenstephan, Germany
*Saccharomyces pastorianus*	3.0277	59	Lager	Lager	Nuremberg, Germany
*Saccharomyces pastorianus*	3.0278	69	Lager	Lager	Nuremberg, Germany
*Saccharomyces pastorianus*	3.0279	120	Lager	Lager	Fürth, Germany
*Saccharomyces pastorianus*	3.0280	128	Lager	Lager	Region Vienna, Austria
*Saccharomyces pastorianus*	3.0281	168	Lager	Lager	Hesse, Germany
*Saccharomyces pastorianus*	3.0282	8-I-4	Lager	Lager	unknown
*Saccharomyces pastorianus*	3.0283	8-J-4	Lager	Lager	unknown
*Saccharomyces pastorianus*	3.0284	8-J-5	Lager	Lager	unknown
*Saccharomyces pastorianus*	3.0285	66/70	Lager	Lager	Dortmund, Germany
*Saccharomyces pastorianus*	3.0286	204	Lager	Lager	Munich, Germany
*Saccharomyces pastorianus*	3.0813	PI BA 124	Lager	Lager	North Rhine-Westphalia, Germany

All yeast strains obtained from the yeast supplier had been cheeked by the guidelines for an accredited laboratory in accordance to the DIN EN ISO/IEC 17025/2005 by the provider. The different yeast strains were tested by MALDI-TOF MS and compared to database entries of Bruker Daltonics (6903; Maldi Biotyper 3 Version 6.0.0.0) and to in-house projects (744). All strains belonged to *S*. *cerevisiae*, *S*. *pastorinanus* or *S*. *cerevisiae* var. *diastaticus* (species identification shown in Table 1).

### Cultivation

#### General

Yeast strains from BLQ were stored in glycerol-stock-media (11 g/L sodium glutamate monohydrate (Carl Roth GmbH & Co. KG, Karlsruhe, Germany), 16 g/L lactose monohydrate (Sigma Aldrich, Darmstadt, Germany), 1 g/L agar (Carl Roth GmbH & Co. KG, Karlsruhe, Germany), 0.1 g/L ascorbic acid (Carl Roth GmbH & Co. KG, Karlsruhe, Germany), 120 g/L 99.5% glycerol (Gerbu, Heidelberg, Germany) and 1 L tap water) at -80°C. For the preparation of the yeast collection, 2 colonies from every strain were inoculated across the entire malt extract agar plates (ME) (20 g/L malt extract (AppliChem GmbH, Darmstadt, Germany), 2 g/L peptone ex soya (Carl Roth GmbH & Co. KG, Karlsruhe, Germany), 15 g/L agar; pH 5.6 (± 0.1)) at 30°C for 2 to 3 days. Subsequently, plates were overgrown with yeast and resuspended in 6 ml of glycerol-stock media. Finally, the suspension was transferred in a 15-ml-tube and stored over night at 4°C. The next day, twice 1.8 ml of the suspension media was transferred in cryogenic tubes to get one working tube and one backup. The tubes were stored at -80°C.

The yeast strains were grown on YPG (5 g/L yeast extract (Carl Roth GmbH & Co. KG, Karlsruhe, Germany), 10 g/L tryptone/peptone ex casein, granulated (Carl Roth GmbH & Co. KG, Karlsruhe, Germany), 20 g/L glucose (Merck, Darmstadt, Germany) and for agar plates 15 g/L agar; pH 6.5 (± 0.1)) at 30°C for 2 to 3 days. Medium was sterilized at 121°C for 15 min. Sugar was sterilized separately and added to the media under a sterile bench.

#### Cultivation of yeasts for database creation

A single colony from the agar plate was picked and inoculated on YPG agar plates for 2 to 3 days at 30°C. From the second plate (working plate), a colony was used to inoculate 15 ml YPG media in 50 ml flasks closed with cotton plugs and aerobic incubated at 30°C for 18 h on a WisML02 rotary shaker with 180 rpm (Witeg Labortechnik GmbH, Wertheim, Germany). After incubation, the samples were prepared for the MALDI-TOF MS analysis and recorded for an in-house database called “Brewing yeast”.

#### Cultivation of strains for bioinformatics analysis

The inoculation of the testing strains was done and additionally modified according to Usbeck, Wilde [36]. A single colony from the agar plate was picked and inoculated on YPG agar plates for 2 to 3 days at 30°C. From the second plate (working plate), a colony was used to inoculate 15 ml YPG media in 50 ml flasks closed with cotton plugs and aerobic incubated at 30°C overnight on a rotary shaker with 180 rpm. After the incubation in YPG media, 1% of the pre-culture was propagated to another 50-ml flask containing 15 ml of YPG media and incubated at 30°C for 18 h on a rotary shaker with 180 rpm. The working plate was used for 4 to 5 days. After incubation, the samples were prepared for the MALDI-TOF MS analysis.

### Analyzing brewing yeasts by MALDI-TOF MS

A volume of 1 ml of each sample was centrifuged (2 min, 13.000 rpm) twice and supernatant removed. The yeast pellet was subsequently resuspended in 300 μl ultra-pure water (J.T. Baker, Deventer, the Netherlands) followed by 5 min mixing. Afterwards 900 μl absolute ethanol (VWR, Fontenay-sous-Bois, France) was added to the suspension and mixed for the same time. After centrifugation, the supernatant was discarded and the pellet air dried for 30 min. Subsequently, proteins were extracted with 50 μl 70% formic acid (Sigma Aldrich, Darmstadt, Germany) and 5 min mixing. 50 μl acetonitrile (ACN) ((Carl Roth GmbH & Co. KG, Karlsruhe, Germany)) was added and the sample mixed again for the same time. After centrifugation, 1 μl of the supernatant was spotted on a MALDI steel-target (Bruker Daltonics, Bremen, Germany), dried in a fume cupboard and overlaid with 1 μl of alpha-cyano-4-hydroxy- cinnamic acid (CHCA for MALDI-TOF MS ≥99% (HPLC)) (Sigma Aldrich, Darmstadt, Germany), prepared with a final concentration of 10 mg/ml (50.0% ACN, 2.5% trifluoroacetic acid (TFA) (Sigma Aldrich, Darmstadt, Germany)) and dried as well.

Mass spectra were generated by a Microflex LT MALDI-TOF MS (Bruker Daltonics, Bremen, Germany) which was equipped with a nitrogen laser (λ = 337 nm) at a laser frequency of 60 Hz operating in linear positive ion detection mode under MALDI Biotyper 3.0 Realtime classification (RTC) (Bruker Daltonics, Bremen, Germany) and FlexControl 3.4 (Bruker Daltonics, Bremen, Germany). The mass range covers an area from 2 to 20 kDa at a voltage of 20.0 kV (ion source 1), 16.80 kV (ion source 2), 6.00 kV (lens) and 2939 kV (linear detector). The laser power was adjusted between 35 to 40% with an offset of 48%. For each spectrum, 240 single spectra, recorded by 40-shot steps from random positions of the target spot, were summarized.

The calibration and validation of MALDI-TOF MS was performed once a week with a bacterial test standard (BTS) (Bruker Daltonics, Bremen, Germany) based on modified *Escherichia coli*. The BTS was resuspended in 100 μl organic solvent (50.0% ACN, 2.5% TFA), stored at -20°C and used till the score-value of this standard was below 2.4 as suggested by the manufacturers.

For database entry, the extraction of a yeast strain was laid on vertical target columns, for example 1 μl sample was spotted per position from A1 to H1 and measured 3 times to obtain 24 spectra per strain. As explained by Bruker Daltonics the evaluation of the main spectra (MSP) was performed by FlexAnalysis 3.4 (Bruker Daltonics, Bremen, Germany), which is a package of Bruker Compass 1.4 (Bruker Daltonics, Bremen, Germany). Afterwards the evaluated spectra were loaded in the in-house database “Brewing yeasts” in MALDI Biotyper 3.0.

For bioinformatics analyses, ten biological replicates along with technical triplicates were recorded on ten different days to obtain 30 spectra per strain. The quantity of replicates covers the variety of peak intensities and mass to charge deviation (600 ppm). Samples were laid in horizontal rows on the MALDI-steel target. All recorded spectra are found within the supporting information covered from S1 File to S18 File. Furthermore, the table in S3 Table and text in S1 Text explains the raw data and the archiving of the files.

### Identification of single spectra and evaluation of database

The 30 single spectra of each brewing yeast strain (Table 1) were identified offline with the brewing yeast database by the MALDI Biotyper 3.0 software. The first matches were taken in count to analyze strain or ecotype (beer type) hits. These results were compared to the actual application of the brewing experience (Table 1).

### Data analysis

The exportation of the recorded mass spectra using FlexAnalysis 3.4 (Bruker Daltonics, Bremen, Germany) and octave-software for pre-processing and data calculations were realized according to Schott, Behr [39]. Based on an open sharedoot computer cluster (ATIX; http://opensharedroot.org) using Mass Spectrometry Comparative Analysis Package (MASCAP) [40], which was implemented in octave software (https://www.gnu.org/software/octave/).

The data analyses were performed based on similarity calculations like Euclidean distance or normalized dot-product for the comparison of recorded mass spectra. Two different approaches were used to analyze the brewing yeasts sub-proteome fingerprints. The first one was to compare the mass spectra 52 brewing yeast strains to each other by a high-throughput multidimensional scaling (HiT-MDS) (http://dig.ipk-gatersleben.de/hitmds/hitmds.html) and hierarchical cluster analysis. The second approach was to compare only the top-fermenting and *S*. *cerevisiae* var. *diastaticus* strains by HiT-MDS with Voronoi calculation, because of the variety within this strains and a discriminant analysis of principal components (DAPC).

MDS is a data processing method suitable for addressing several analytical purposes: (i) for dimension reduction of vector data, providing a nonlinear alternative to the projection to principal components; (ii) for the reconstruction of a data dissimilarity matrix of pairwise relationships in the Euclidean output space; (iii) for conversion of a given metric space, such as data compared by Manhattan distance, into Euclidean space and (iv) for dealing with missing data relationships using zero force assumption [41]. It has been predominantly used as a tool for analyzing proximity data of all kinds. Most for all, MDS serves to visualize such data making them accessible to the eye of the researcher. For example, the distance between two points represent the correlation of the respective variables. As all variables are non-negatively intercorrelated, it is particularly easy to interpret this MDS configuration: The closer two points, the higher the correlation of the variables they represent [42]. The visualization of relationships between different data records can be obtained by reconstructing these relationships as pairwise distances in the usual Euclidean 2D plane or 3D space [41]. A HiT-MDS is an optimized version for rapid distance reconstruction, based on correlations of distances between input and output space (http://dig.ipk-gatersleben.de/hitmds/hitmds.html). The HiT-MDS is mentioned within the scientific work as MDS.

In order to decrease the complexity of the diagram from every brewing yeast strain, the 30 single spectra were summarized to one consensus spectrum for MDS. Summarized spectra were compared subsequently to each other for similarity and plotted in a 2D map. At the end of the calculation the reconstruction quality from 0 to 1 (1 is prefect reconstruction) was displayed. This was performed for all top- and bottom-fermenting as well as *S*. *cerevisiae* var. *diastaticus* strains.

Furthermore, the summarized spectra were evaluated by a hierarchical cluster analysis by an in-house software based on MASCAP [40]. The calculation of the cluster analysis was accomplished to weighted pair group method with averaging (WPGMA) [43, 44] and a normalized dot-product, which determine the similarity between recorded mass spectra and is explained in Frank, Bandeira [45].

The second approach was to analyze 32 top-fermenting and 7 *S*. *cerevisiae* var. *diastaticus* strains together by MDS (http://dig.ipk-gatersleben.de/hitmds/hitmds.html). The MDS was done likewise with summarized spectra and Voronoi triangulation [46] was performed for dividing the yeast strains in groups named to the beer types. The Voronoi triangulation is based on a decomposition of metric space by distances between sets of points [46], in our case beer types, which are divided into cells each containing one focus, marked with the beer type name in capitals. It is included in octave (https://www.gnu.org/software/octave/).

In addition, the 39 selected yeast strains were analyzed by DAPC using the *adegenet* package (2.0.1) for using RStudio software [47]. DAPC seeks synthetic variables, the *discriminant functions*, which show differences between groups as best as possible while minimizing variation within clusters [47]. The raw data were transformed using PCA, which is followed by *k*-means algorithm with increasing values of *k* to identify the optimal number of cluster. Different clustering solutions are compared using Bayesian Information Criterion (BIC). Ideally the optimal clustering solution should correspond to the lowest BIC and is visualized by an elbow in the curve. After choosing a number of clusters the discriminant analysis was performed to obtain a barplot of eigenvalues, and finally a scatterplot was obtained which represents the individuals as dots and the groups as inertia ellipses. Furthermore, it is possible to visualize groupings by a histogram and the main peaks responsible for the separation in a loading plot.

All single spectra of the 39 yeast strains (n = 1170) were analyzed by this tool to obtain a scatterplot to visualize beer types as inertia ellipses, histogram and loading plot.

Visualization of spectra from chosen strains were realized according to Schott, Behr [39] to distinguish and compare the brewing yeasts on the recorded sub-proteomic pattern within an area from 2000 to 12000 Da.

For all bioinformatics analysis a mass range from 2000 to 20000 Da is taken into account.

## Results

### Differentiation of all 52 brewing yeast strains by bioinformatics approaches

The 52 brewing yeast strains were recorded by MALDI-TOF MS on 10 different days with technical triplicates to get 30 spectra per strain. Overall 1560 single spectra were used for bioinformatics analyses.

**Fig 1:**
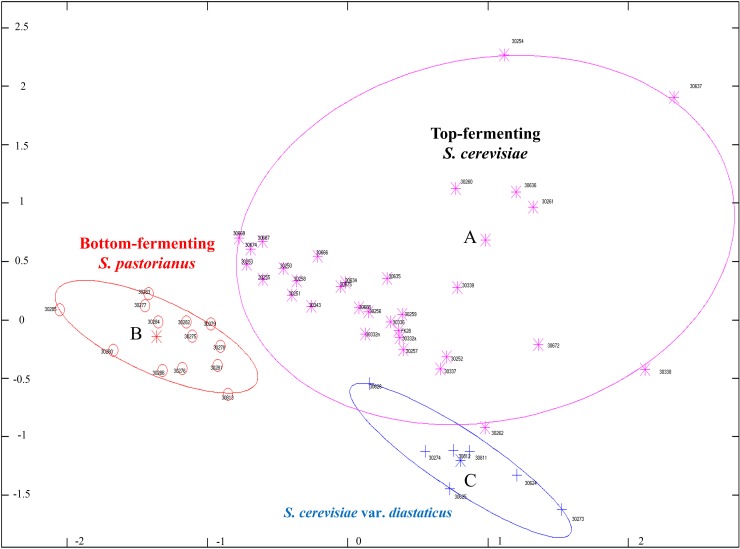
**Multidimensional scaling (MDS) of 52 brewing yeast strains separated in top- (A) and bottom-fermenting (B) as well as S. cerevisiae var. diastaticus (C).** All labels represent the mean spectra of 30 single spectrums of each strain; strains are presented with different ID’s according to Lehrstuhl of Technische Mikrobiologie (TMW); top-fermenting are depicted in purple colored stars, a purple ellipse symbolized the group and the letter A is the center; bottom-fermenting are depicted in red colored circles, a red ellipse symbolized the group and the letter B is the center; *S*. *cerevisiae* var. *diastaticus* are depicted in blue colored crosses, a blue ellipse symbolized the group and the letter C is the center; The x and y axis represent the distances from every label to each other; ans = 0.95132; n = 52.

The separation of top-, bottom-fermenting brewing yeasts and *S*. *cerevisia* var. *diastaticus* strains by MDS is shown in Fig 1. The separation of top- and bottom fermenting yeast and *S*. *cerevisia* var. *diastaticus* strains was performed using MDS. The analyzed mass spectra were separated in three groups (A, B, C). The data was labeled with the strain ID and the fermentation behavior. Group A harbored spectra of top-fermenting *S*. *cerevisiae* strains and formed the biggest section. Spectra of bottom-fermenting *S*. *pastorianus* strains were included in group B and placed on the left side of the MDS. The seven strains of the variety *S*. *cerevisiae* var. *diastaticus* formed group C and are below of group A. A separation between group “B” to “A and C” is recognized as well as a differentiation of “A” to “C” in Fig 1. Two outliers were identified and visualized. *S*. *cerevisiae* TMW 3.0262, belonging to group A, shows similarities to *S*. *cerevisiae* var. *diastaticus* and thus is next to the ellipse of group C. *S*. *cerevisiae* var. *diastaticus* TMW 3.0628 in A, belonging to group C, shows strong similarities to top-fermenting S. cerevisiae and thus is even inside the ellipse of group A.

**Fig 2:**
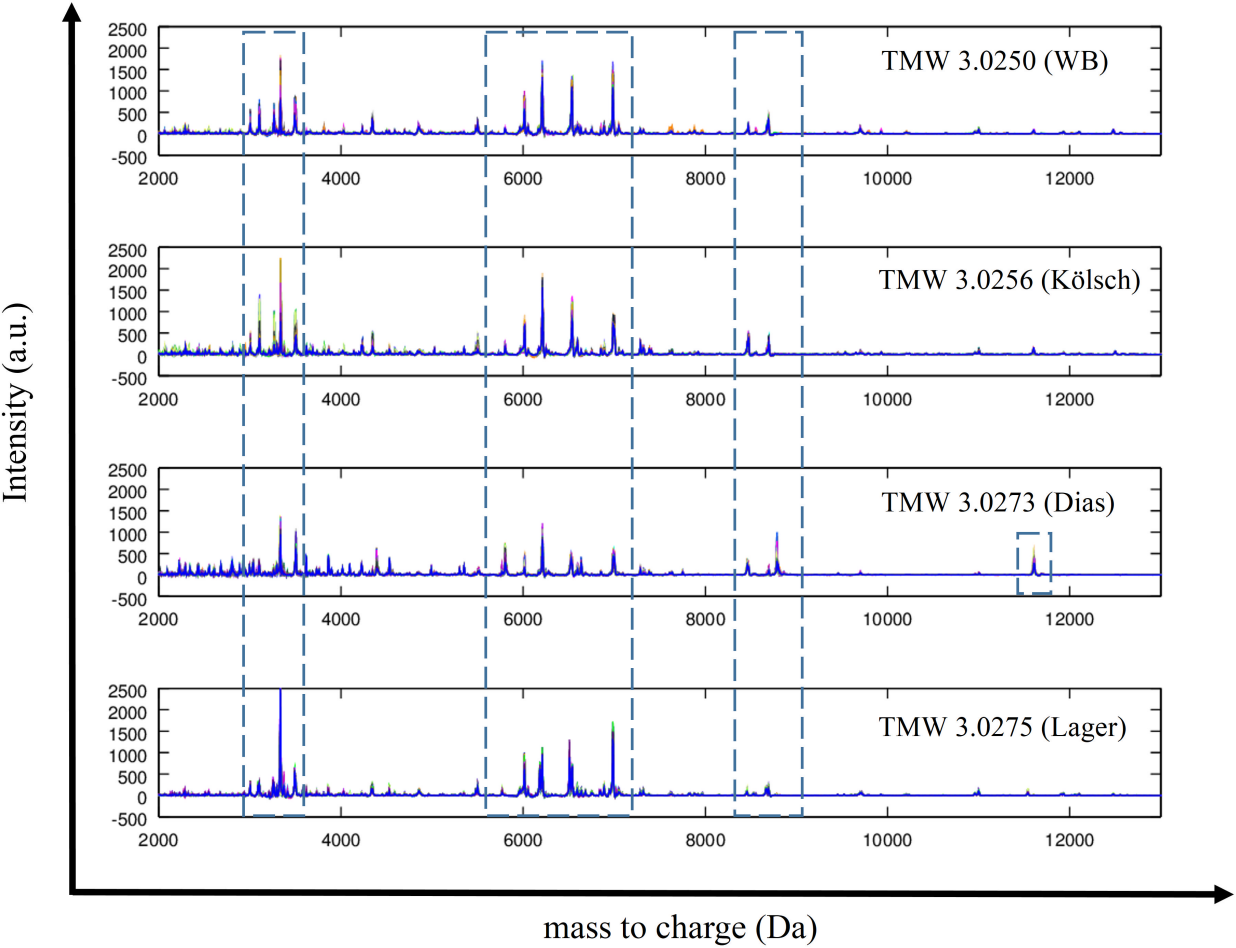
Stacked spectra of four different strains. 30 single spectra of each strain were summarized to one mean spectra; y-axis represent the intensity (= Int) of the recorded peaks; x-axis show the mass to charges from 2000 to 12000 Da; the ID of each strain is placed on the right sight of every spectra with the code TMW (Technische Mikrobiologie Weihenstephan); beer types are written in brackets (WB = wheat beer; Dias = *S*. *cerevisiae* var. *diastaticus*); blue boxes with dotted lines figure out peak differences.

Spectra of four brewing yeasts (of the top- and bottom fermenting as well as *S*. *cerevisiae* var. *diastaticus* yeast) were stacked and visually compared (Fig 2). The illustration figured out the major peak differences and the dissimilarity within the species. The dotted bars in the peak spectrum display main differences within the mass to charge ratio of 2000 to 12000 m/z. In the area of 6000 to 7000 Da major peak differences are visualized. Furthermore, TMW 3.0273 has a unique sub-proteomic peak around 11800 Da, which did not occur in any other species.

**Fig 3:**
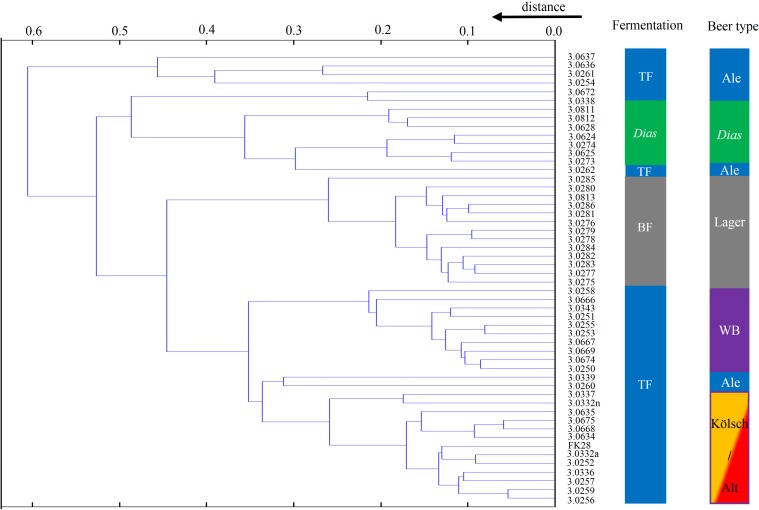
Cluster analysis of 52 yeast strains which are displayed in a hierarchical dendrogram and labeled to fermentation and beer type. Every ID represent the mean spectra of 30 single spectra per strain; the fermentation type is labeled to top-fermenting (= TF; blue), bottom-fermenting (= BF; grey) and *S*. *cerevisiae* var. *diastaticus* (= *Dias*; green); beer types are illustrated to Ale (blue), *S*. *cerevisiae* var. *diastaticus* (= *Dias*; green), Lager (grey), wheat beer (= WB; purble), Kölsch (yellow) and Alt-beer (= Alt; red); the distance is instructed from 0.0 (high similarity) to 0.6 (large distinction).

A hierarchical cluster analysis was performed to separate the brewing yeasts in a dendrogram (Fig 3). Thereby, 52 yeast strains, outlined with the TMW-number, were clustered and additionally labelled according to the fermentation and the beer type. Considering the fermentation type, three different labels are present: bottom-fermenting (BF), *S*. *cerevisiae* var. *diastaticus* (Dias) and top-fermenting (TF) whereby TF is separated in three parts. The labeling according to the beer type is similar to the labeling according to the fermentation type. However, the main difference is that top-fermenting brewing yeast strains are parted in respect to the current beer type. The wheat beer (WB; purple) strains are separated in a single cluster apart from all other top-fermenting strains. The separation of Kölsch / Alt beer strains from each other is not possible (mix of red and orange). Ale strains are more heterogeneous and are harbored in six groups. The first cluster is parted in three sub-clusters containing six Ale strains such as TMW 3.0637, 3.0636, 3.0261, 3.0254, 3.0672 and 3.0338. The Ale strain TMW 3.0262 is clustered as an outlier to the *S*. *cerevisiae* var. *diastaticus* strains and furthermore the Ale strains TMW 3.0260, 3.0339 are related to the Kölsch / Alt beer strains.

### Comparison of BLQ-strains to database

The comparison of the BLQ-strains to their own database entries is shown in S1 Table and percentage hit-rate on strain-level and beer type in S2 Table. 1560 single spectra (30 spectra per strain) were recorded by MALDI-TOF MS and compared to 52 database entries which are divided in different beer types: 32 top-fermenting (10 wheat beer, 9 Ale, 8 Alt-beer, 5 Kölsch), 7 *S*. *cerevisiae* var. *diastaticus* and 13 bottom-fermenting (11 flocculent and 2 powdery) brewing yeast strains. *S*. *pastorianus* and *S*. *cerevisiae* were fully separated (100%) to *S*. *cerevisiae* subsp. *diastaticus*. This is in accordance with the results of bioinformatics analyses (Figs 1 and 3). Furthermore, nearly all wheat beer strains (300 single spectra) were classified to the area of wheat beer and showed an average hit rate to this type of 99.33%. 91.48% of brewing yeasts belonging to the Ale type formed the Ale area with one exception. TMW 3.0668 (TUM 513) is used as an Ale strain by brewers (Table 1). Nevertheless, it was classified by MALDI-TOF MS to the Alt / Kölsch region and was labeled for further analysis as an Alt-beer strain. Similarity, TMW 3.0254, which was originally classified as a Kölsch strain (Table 1), was classified by MALDI-TOF MS as an Ale-strain. This strain was re-labeled for additional analysis as an Ale-strain. Regarding the strains of Alt and Kölsch and considering the average hits of 83.75% (Alt) and 40.00% (Kölsch), there is no clear separation of these beer types. The 7 strains of *S*. *cerevisiae* var. *diastaticus* formed a single group and showed an average match of 100% to the ecotype level of *Diastaticus*. The bottom-fermenting yeast strains are presented with a hit rate of 99.39% (flocculent) and 35% (powdery).

An overall average hit rate of 37.88% to strain level was achieved. Only the strains TMW 3.0338 and TMW 3.0339 matched 100% to their database entries.

### Characterization of top-fermenting and *S*. *cerevisiae* var. *diastaticus* strains by bioinformatics

**Fig 4:**
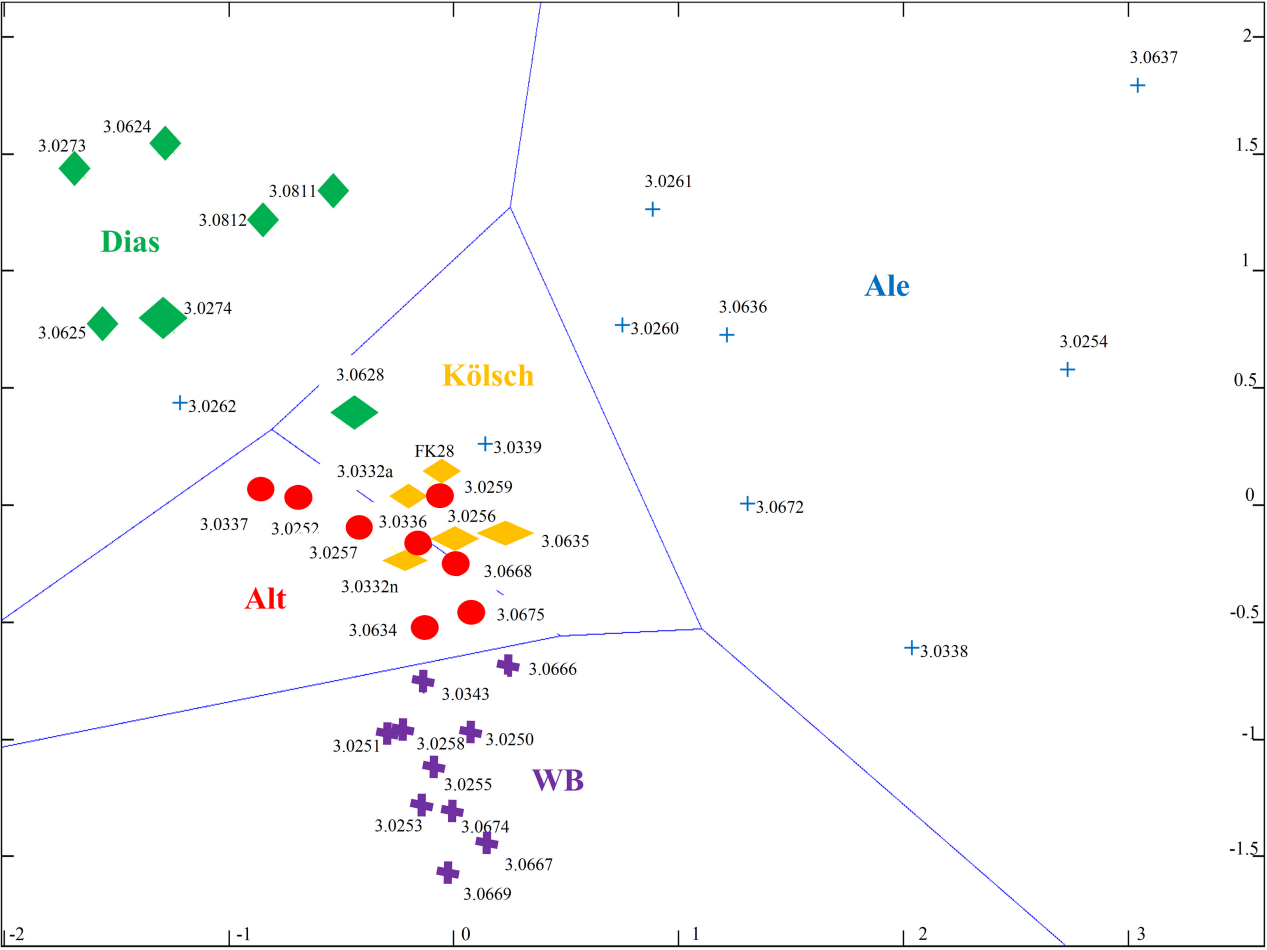
Multidimensional scaling (MDS) containing 32 top-fermenting brewing yeast and seven S. cerevisiae var. distaticus strains divided in different beer types by MALDI-TOF MS. Beer types are labeled in the center of each group and colored (WB = wheat beer (purpble crosses); Ale (blue crosses); Kölsch (yellow rhombuses); Alt = Alt-beer (red circles); S. *cerevisiae* var. *diastaticus* = *Dias* (green diamonds)); ID represents the number of Technische Mikrobiologie Weihenstephan (TMW); x-axis and y-axis displayed the distance; ans = 0.94807; n = 39.

The separation of different beer types of the top-fermenting yeasts and *S*. *cerevisiae* var. *diastaticus* was performed (Fig 4). For 39 brewing strains, a similarity computation was done to visualize differences between the strains in a 2D map by MDS with Voronoi triangulation (blue lined). If the distances between the labels are big, the more different the MALDI patterns (based on one mean spectrum summarized 30 spectra per strain) will be.

The 10 WB-strains were distinguished from the *S*. *cerevisiae* var. *diastaticus* group (Dias; green diamond), which is located on left top sight of the 2D map. The differentiation of Alt (red circles) and Kölsch (yellow rhombus) was not achieved. The Ale strains show a high degree of dissimilarity (blue cross) which is displayed by the wide group on the right corner of the MDS. Furthermore, they clearly separate to the other beer types. Nevertheless, strain TMW 3.0262 shows similarities to the *Diastaticus* group. In parallel, TMW 3.0668 is dropped to the Alt / Kölsch region as well to the database comparison (supplementary). The *S*. *cerevisiae* var. *diastaticus* strain TMW 3.0628 and the Ale strain TMW 3.0339 are placed to the region of Kölsch like.

**Fig 5:**
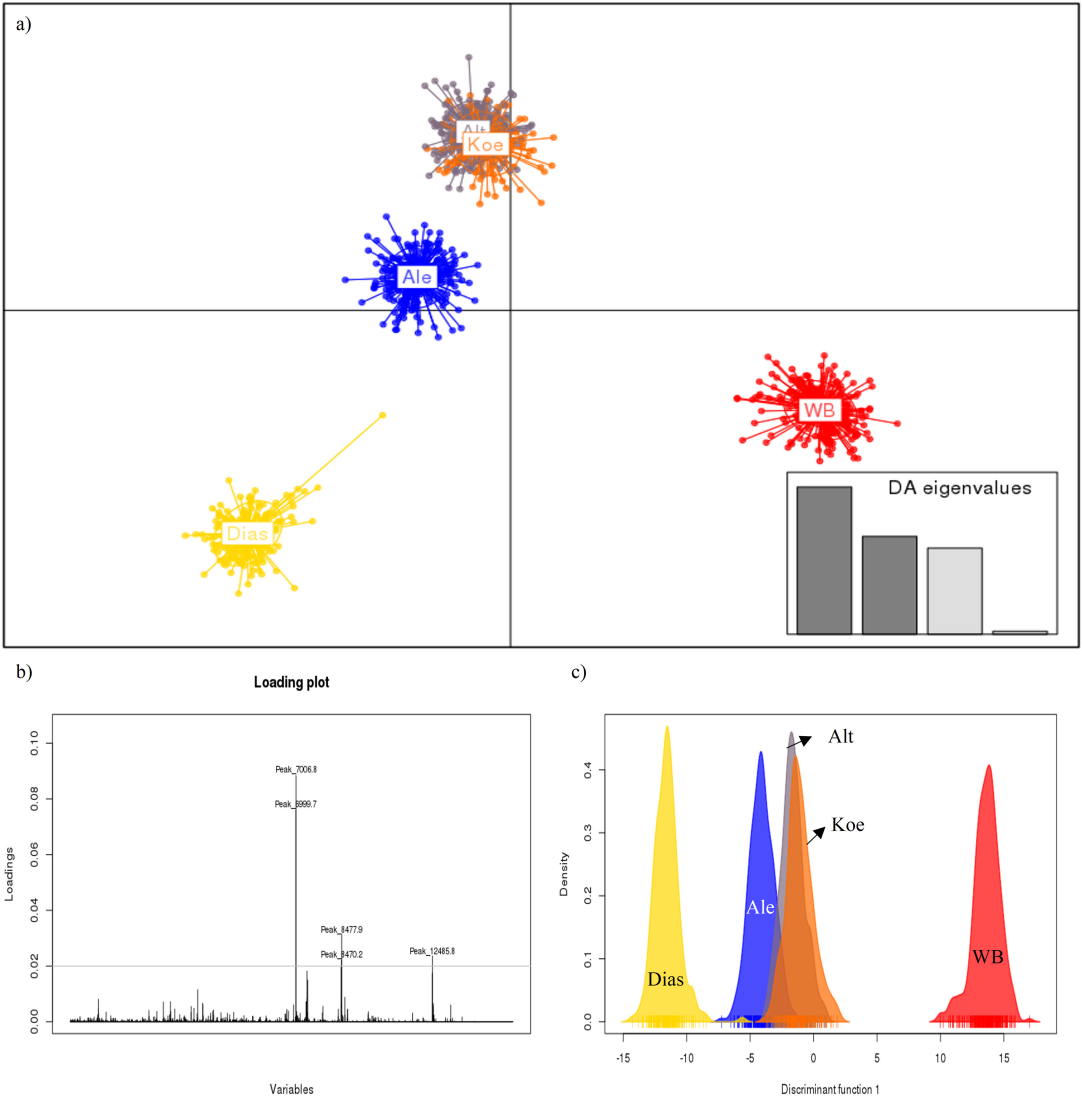
**a) Discriminant analysis of principal components (DAPC) of top-fermenting brewing yeasts and *S*. *cerevisiae* var. *diastaticus*.** 1170 single spectra are illustrated and labeled by dots; WB = wheat beer (red), Koe = Kölsch (orange), Alt = Alt-beer (grey), Ale (blue), Dias = *S*. *cerevisiae* var. *diastaticus (yellow)*. **b) Visualizing of major peaks which are responsible for the separation by a loading plot. c) Histogram of the recorded spectra and labeled to different beer types.** WB = wheat beer (red), Koe = Kölsch (orange), Alt = Alt-beer (grey), Ale (blue) and Dias = *S*. *cerevisiae* var. *diastaticus* (yellow).

The calculation of discriminant analysis of principal components (DAPC) with a loading plot and histogram of all single spectra is shown in Fig 5 and is supported by the cluster analysis and MDS. All recorded spectra are clustered in groups as ellipses and labeled according to the beer types. Within five clusters of beer types that were distinguished, the wheat beer cluster (WB; red) and the *S*. *cerevisiae* var. *diastaticus* (Dias; yellow) display a clear separation from other groups (Fig 5A). The loading plot that is shown in Fig 5B summarizes all single spectra of the yeast strains and represents those peaks, which are responsible for the separation. The highest loadings are achieved at 6999.2 Da and 7006.8 Da. The separation is supported by the histogram for discriminant axis/funtion (Fig 5C). The histogram displays the similarity of Alt (grey) and Kölsch (orange) and shows also the isolation of wheat beer (red) and *diastaticus* (yellow).

**Fig 6:**
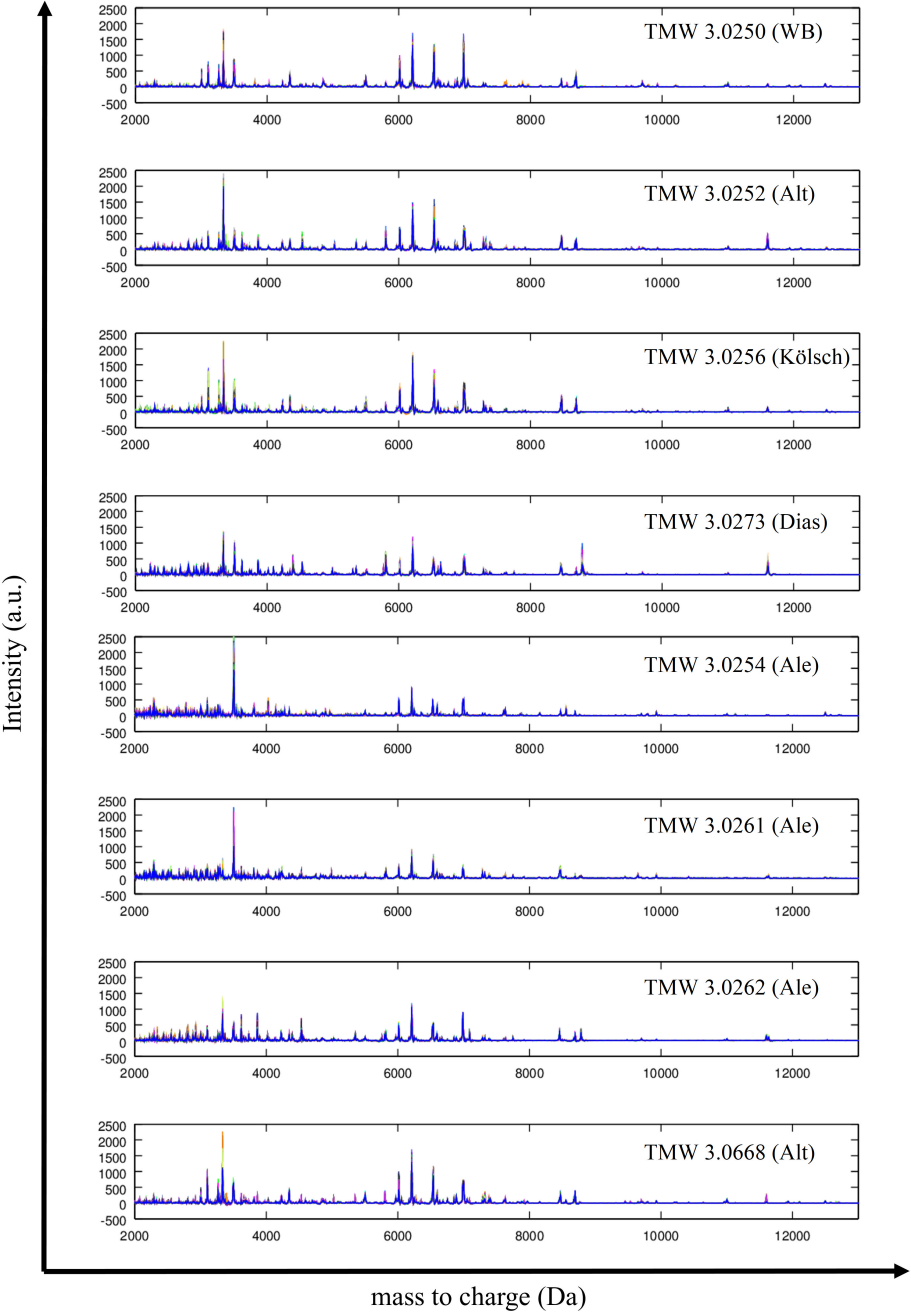
Stacked spectra of eight different strains. 30 single spectra of each strain were summarized to one mean spectra; y-axis represent the intensity (= Int) of the recorded peaks; x-axis show the mass to charges from 2000 to 12000 Da; the ID of each strain is placed on the right sight of every spectra with the code TMW (Technische Mikrobiologie Weihenstephan); beer types are written in brackets (WB = wheat beer; Alt = Alt-beer; Dias = *S*. *cerevisiae* var. *diastaticus*).

The visualization of 8 single spectra overlays of TMW 3.0250 (WB), TMW 3.0252 (Alt), TMW 3.0256 (Kölsch), TMW 3.0273 (Dias), TMW 3.0254 (Ale), TMW 3.0261 (Ale), TMW 3.0262 (Ale) and TMW 3.0668 (Alt) is illustrated in Fig 6 and demonstrates the differences of MALDI fingerprints of different ecotypes. The sub-proteome of TMW 3.0252 and 3.0256 are similar to each other but slightly differ in the intensity of some peaks. The protein profile of TMW 3.0250 shows a single peak with a high intensity around 7000 Da which was found in all WB-strains. The sub-proteome of the variety *diastaticus* represented by TMW 3.0273 displays several peaks between 3000 to 5000 Da. Furthermore, a single peak around 9000 Da and 11800 Da was detected, respectively. The sub-proteome of TMW 3.0668 visually shows more similarities to TMW 3.0252 and 3.0256 than to the other Ale-strains. Nevertheless, TMW 3.0254 a Kölsch strain to brewer experience and an Ale-strain to MALDI-TOF MS showed more similarities to TMW 3.0261 as to TMW 3.0256 strains. Those findings are reflected in Fig 3, Fig 4 and S1 Table.

## Discussion

In this work proof of concept of the discriminatory power of MALDI-TOF MS was provided towards the application potential of *Saccharomyces* yeasts. Therefore, a sub-proteome spectra database was established for brewing yeasts (top- and bottom-fermenting) of the major beer types, and for the beer spoilage microorganism *S*. *cerevisiae* var. *diastaticus*. This database allows the assignment of brewing yeast strains not only to their respective species but also to a specific beer type of preferential use.

The use of MALDI-TOF MS as a tool to identify microorganism on a species level is a common application by clinical samples [32, 48]. However, there are a lot of approaches to apply this method for the classification of food fermentation microbiota and starter cultures [49, 50], food spoilage microbiota [51] or beverage spoiling strains [31, 52]. This method demonstrates the potential to investigate the influences of different stress responses on microorganisms in different media composition. In some studies it has been shown that different stress qualities like acid and hop shock [53], oxidative stress, starvation stress or different metabolic status influence the sub-proteome [39, 54]. Furthermore, it could also be used to classify microorganisms into different groups and separate strains to a specific ecotype namely their spoilage potential. *L*. *brevis* strains, which are strong and moderate beer spoilers could be separated from weak spoilers by MALDI-TOF MS, because of their sub-proteome [29].

The comparison of 52 brewing yeast strains investigated in this work to the generated “brewing yeast” database entries showed a 100% separation to species level. Bottom-fermenting *S*. *pastorianus* strains such as TMW 3.0275 distinguished from all *S*. *cerevisiae* strains, because of the sub-proteomic pattern (Fig 2).

The bottom-fermenting yeasts are capable to ferment the trisaccharide raffinose fully, since they use the enzyme melibiase, which splits the disaccharide melibiose to galactose and glucose [13]. Another property of bottom-fermenting yeast is the ability to flocculate. This complex process depends on the expression of specific flocculation genes such as FLO1, FLO5, FLO8 and FLO11 [55]. Even though we recorded different bottom-fermenting yeast strains according to their flocculation behavior, a clear separation within this species could not be attained. Nevertheless, for the commercial usage it is important to differentiate between top- and bottom-fermenting brewing yeasts, which was feasible.

Strain level identification of all 52 brewing yeasts could not be achieved because of their highly similar spectra. Species and sub-species typing was observed for wine yeasts [36] as well. MALDI-TOF mass spectral profiles of *S*. *cerevisiae* strains from different origins showed as well various sub-proteomic patterns and brewing yeast strains distinguished from other commercial yeast strains which was observed by Moothoo-Padayachie, Kandappa [37]. However, the fingerprints differed with respect to the beer types for which these strains are regularly used. Because of that, strains, which are used for the production of wheat beer, could be differentiated from all the other top-fermenting yeast strains (Figs 2 and 3), which are employed for other beer types. Wheat beer strains showed a unique peak at 6985 Da, fingerprints of the other *S*. *cerevisiae* strains displayed a double peak in this area at 6986 Da and at 7001 Da. It seems that those small and big proteins contains variations, and it may be easier to detect variations in small proteins than in those of higher molecular weight, which are jointly responsible for the differentiation from wheat beer strains to Ale, Alt and Kölsch strains. Wheat beer is, particularly in Germany [56], a top-fermented specialized beer and differs from other beer types within the sensory impressions such as phenolic or estery flavor [13]. The wheat beer strains used for this beer type not only differ on sub-proteomic level to the other brewing yeast strain. Gonçalves, Pontes [27] found out that based on genomic analysis wheat beer strains could be differentiated from all the other brewing yeast strains like English-Irish Ale and German Alt-Kölsch. Moreover, Gallone, Steensels [28] described the group of wheat beer strains as well by genomic analysis and explained this population structure with a result from a cross between an ale beer strain and a wine strain. The use of Polymerase Chain Reaction-Denaturing High Performance Liquid Chromatography (PRC-DHPLC) for the differentiation of brewing yeast strains showed that the profiles of wheat beer strains were very similar and differ from other yeasts likewise [57].

Besides that, wheat beer strains produce a lot of fermentation by-products. Schneiderbanger, Koob [56] tested five different wheat beer strains on the production of acetate esters and showed correlations to the gene expression of ATF1. PAD1 and FDC1 genes are responsible for phenolic off flavors (POF), which occur by the decarboxylation of ferulic acid and results in 4-vinyl guaiacol (clove flavor) [58, 59]. Wheat beer strains have rather those POF-genes than yeasts related to other beer types, as it is described in Gonçalves, Pontes [27]. This flavor is quite typical for wheat beers [27] and is dependent on the ferulic acid concentration in beer wort [60].

The characteristic property of producing POF and of fermenting starch and dextrin is found in *S*. *cerevisiae* var. *diastaticus* equally [61]. Genes STA1, STA2 and STA3 produce extracellular glucoamylase [62] and hydrolyze alpha-D (1–6) bonds beside alpha-D (1–4) ones [63]. These metabolic and fermentation behavior is characteristic for these variety as it is explained by Andrews and Gilliland [64]. Likewise, it was figured out that *S*. *cerevisiae* var. *diastaticus* strains caused low specific gravities (super-attenuation) and an excessive pressure in bottled beer. This is due to a rapid fermentation and formation of high amounts of carbon dioxide. Therefore, it is important to distinguish top-fermenting *S*. *cerevisiae* strains and beer spoilage yeast *S*. *cerevisiae* var. *diastaticus* by a rapid method like MALDI-TOF MS which could be achieved.

A differentiation between Alt and Kölsch could not be achieved (Figs 3 and 5A and 5C) and was explained by recorded spectra (Fig 6). The mass spectra showed small deviation to each other and might be caused by the different geographical use of these yeast strains. Over the years, brewers from Cologne or Dusseldorf likely used the same group of brewing yeasts and the major difference of these beer types result from other resources [65]. In fact, brewing yeasts related to these beer types can be used for Alt-beer as well as for Kölsch production and may be looked at as one group as proposed by Gonçalves, Pontes [27] with “German Alt-Kölsch”.

The wide variety of Ale strains can also be explained by the sub-proteomic patterns (Fig 6), which differ between a high (TMW 3.0262) and a small (TMW 3.0621) amount of low molecular sub-proteins and furthermore strongly distinguishes them from wheat beer strains (TMW 3.0250). Based on the dendrogram and MDS, several yeasts related to this beer type cluster are outliers and are assigned to other beer types (Figs 3 and 4). Especially TMW 3.0668 is clustered to Alt-beer. Actually, it is classified by the experience of brewer as an Ale yeast. The spectra of TMW 3.0668 (Fig 6) showed higher similarities to the spectrum of German Alt-Kölsch strains then to the Ale yeasts. Gonçalves, Pontes [27] explained that some strains, which were used for fermenting Alt-beer in Germany, are exported to countries like the USA where they are used for the fermentation of other beer-types and could subsequently be assigned to a new beer type such as Ale. This generally suggests that assignment to a beer type need not necessarily restrict the use of a specific yeast exclusively for that beer type.

Different studies showed the possibility to identify and characterize yeast of the genus *Saccharomyces* by DNA-based methods, which resulted in various groupings based on their origin or application. Legras, Merdinoglu [26], Gonçalves, Pontes [27] and Gallone, Steensels [28] investigated independently the genomic background of *Saccharomyces* strains from different geographical origins and applications and they were able to cluster them to wine and cider, rum and distillery, bread, lager, ale and other groups. Some of the brewing yeast strains, which were used in this study, were analyzed via Delta-PCR and rDNA IGS2_314 genetic fingerprints by the yeast center of Weihenstephan. A grouping of the brewing yeast was detected with relation to a specific application (personal communication with Mathias Hutzler, BLQ, Technische Universität München, Germany).

With the establishment of this MALDI-TOF MS spectra database proof of concept is provided of the discriminatory power of this technique to classify brewing yeasts into different major beer types in a rapid, easy way, and focus brewing trails accordingly. It can be extended to yeasts for specialty beer types and other applications including wine or baking.

## Supporting information

S1 Table Identification of 1560 recorded spectra, which are compared to 52 brewing yeast database entries. The hit rates (%) of the tested strains are displayed to the database entries and show whether hits on strain (green squares) or ecotype-level (all yeast strains of an appointed beer type); a hit rate of 100% displays a total strain identification; database entries are displayed on the top of the table with the ID of the Technische Mikrobiologie Weihenstephan (TMW) and organized to beer types, which are displayed above; on the left side are all recorded strains with the ID of TMW; 30 spectra of each strain were compared to the database entries.
(XLSX)

S2 Table The average hit rate (%) on brewing type and strain level for top-fermenting, bottom-fermenting (flocculent / powdery behavior) and *S*. *cerevisiae* var. *diastaticus*. (XLSX)

S3 Table Raw files archive description. Column “A” represent the archive name and explains in which directory the raw files are; Column “B” represents the sub-folder name for each supplementary file including numbers from one to 52; Column “C” represent the included raw spectra in the relevant directory.
(XLSX)

S1 File MALDI-TOF MS raw files 1–3 of the strains TMW 3.0250, TMW 3.0251 and TMW 3.0252. The directory includes 90 raw files and was zipped to “.zip”; the folder has three strains with 30 spectra per strain; S1 Text explains the raw files with an example and Table 1 represents the yeast strains used within the experiment.
(ZIP)

S2 File MALDI-TOF MS raw files 4–6 of the strains TMW 3.0253, TMW 3.0254 and TMW 3.0255. The directory includes 90 raw files and was zipped to “.zip”; the folder has three strains with 30 spectra per strain; S1 Text explains the raw files with an example and Table 1 represents the yeast strains used within the experiment.
(ZIP)

S3 File MALDI-TOF MS raw files 7–9 of the strains TMW 3.0256, TMW 3.0257 and TMW 3.0258. The directory includes 90 raw files and was zipped to “.zip”; the folder has three strains with 30 spectra per strain; S1 Text explains the raw files with an example and Table 1 represents the yeast strains used within the experiment.
(ZIP)

S4 File MALDI-TOF MS raw files 10–12 of the strains TMW 3.0259, TMW 3.0260 and TMW 3.0261. The directory includes 90 raw files and was zipped to “.zip”; the folder has three strains with 30 spectra per strain; S1 Text explains the raw files with an example and Table 1 represents the yeast strains used within the experiment.
(ZIP)

S5 File MALDI-TOF MS raw files 13–15 of the strains TMW 3.0262, TMW 3.0273 and TMW 3.0274. The directory includes 90 raw files and was zipped to “.zip”; the folder has three strains with 30 spectra per strain; S1 Text explains the raw files with an example and Table 1 represents the yeast strains used within the experiment.
(ZIP)

S6 File MALDI-TOF MS raw files 16–18 of the strains TMW 3.0275, TMW 3.0276 and TMW 3.0277. The directory includes 90 raw files and was zipped to “.zip”; the folder has three strains with 30 spectra per strain; S1 Text explains the raw files with an example and Table 1 represents the yeast strains used within the experiment.
(ZIP)

S7 File MALDI-TOF MS raw files 19–21 of the strains TMW 3.0278, TMW 3.0279 and TMW 3.0280. The directory includes 90 raw files and was zipped to “.zip”; the folder has three strains with 30 spectra per strain; S1 Text explains the raw files with an example and Table 1 represents the yeast strains used within the experiment.
(ZIP)

S8 File MALDI-TOF MS raw files 22–24 of the strains TMW 3.0281, TMW 3.0282 and TMW 3.0283. The directory includes 90 raw files and was zipped to “.zip”; the folder has three strains with 30 spectra per strain; S1 Text explains the raw files with an example and Table 1 represents the yeast strains used within the experiment.
(ZIP)

S9 File MALDI-TOF MS raw files 25–27 of the strains TMW 3.0284, TMW 3.0285 and TMW 3.0286. The directory includes 90 raw files and was zipped to “.zip”; the folder has three strains with 30 spectra per strain; S1 Text explains the raw files with an example and Table 1 represents the yeast strains used within the experiment.
(ZIP)

S10 File MALDI-TOF MS raw files 28–30 of the strains TMW 3.0332a, TMW 3.0332n and TMW 3.0336. The directory includes 90 raw files and was zipped to “.zip”; the folder has three strains with 30 spectra per strain; S1 Text explains the raw files with an example and Table 1 represents the yeast strains used within the experiment.
(ZIP)

S11 File MALDI-TOF MS raw files 31–33 of the strains TMW 3.0337, TMW 3.0338 and TMW 3.0339. The directory includes 90 raw files and was zipped to “.zip”; the folder has three strains with 30 spectra per strain; S1 Text explains the raw files with an example and Table 1 represents the yeast strains used within the experiment.
(ZIP)

S12 File MALDI-TOF MS raw files 34–36 of the strains TMW 3.0343, TMW 3.0624 and TMW 3.0625. The directory includes 90 raw files and was zipped to “.zip”; the folder has three strains with 30 spectra per strain; S1 Text explains the raw files with an example and Table 1 represents the yeast strains used within the experiment.
(ZIP)

S13 File MALDI-TOF MS raw files 37–39 of the strains TMW 3.0628, TMW 3.0634 and TMW 3.0635. The directory includes 90 raw files and was zipped to “.zip”; the folder has three strains with 30 spectra per strain; S1 Text explains the raw files with an example and Table 1 represents the yeast strains used within the experiment.
(ZIP)

S14 File MALDI-TOF MS raw files 40–42 of the strains TMW 3.0636, TMW 3.0637 and TMW 3.0666. The directory includes 90 raw files and was zipped to “.zip”; the folder has three strains with 30 spectra per strain; S1 Text explains the raw files with an example and Table 1 represents the yeast strains used within the experiment.
(ZIP)

S15 File MALDI-TOF MS raw files 43–45 of the strains TMW 3.0667, TMW 3.0668 and TMW 3.0669. The directory includes 90 raw files and was zipped to “.zip”; the folder has three strains with 30 spectra per strain; S1 Text explains the raw files with an example and Table 1 represents the yeast strains used within the experiment.
(ZIP)

S16 File MALDI-TOF MS raw files 46–48 of the strains TMW 3.0672, TMW 3.0674 and TMW 3.0675. The directory includes 90 raw files and was zipped to “.zip”; the folder has three strains with 30 spectra per strain; S1 Text explains the raw files with an example and Table 1 represents the yeast strains used within the experiment.
(ZIP)

S17 File MALDI-TOF MS raw files 49–51 of the strains TMW 3.0811, TMW 3.0812 and TMW 3.0813. The directory includes 90 raw files and was zipped to “.zip”; the folder has three strains with 30 spectra per strain; S1 Text explains the raw files with an example and Table 1 represents the yeast strains used within the experiment.
(ZIP)

S18 File MALDI-TOF MS raw files 52–54 of the strain FK28. The directory includes 30 raw files and was zipped to “.zip”; the folder has one strain with 30 spectra; S1 Text explains the raw files with an example and Table 1 represents the yeast strain used within the experiment.
(ZIP)

S1 Text Explanation of raw files which includes an example. (DOCX)
