# Supplementary material for: MALDI-TOF MS typing enables the classification of brewing yeasts of the genus Saccharomyces to major beer styles
Source: PLoS One. 2017 Aug 9;12(8):e0181694. doi: 10.1371/journal.pone.0181694 (PMC5549903; doi:10.1371/journal.pone.0181694)
Supplement: S1 Text — (DOCX) [file pone.0181694.s022.docx]

**Explanation of raw files**

All raw_files have the same amount of labels and differentiate within the beer types, strain number and fermentation behavior. An example is presented and every label is explained. Those explanation counts for every sample. Every label is separated with a hyphen (-) and the end of each data show the date of recording and position number of the MALDI-target like “A1”. The numeric list seen below correspond to the labels of each data.

*Example:*

30250-68-OG-WB-WB-YPG-18h-E-E_2015-07-06_1_0_A1_1.dat

1. 30250

🡪 Coding of “Lehrstuhl für technische Mikrobiologie” (TMW 3.0250)

1. 68

🡪 Coding of „Forschungszentrum Weihenstephan für Brau- und Lebensmittelqualität“ (TUM 68)

1. OG / Lag / Dias

🡪 Fermentation behavior; OG = Top-fermenting; Lag = Lager (bottom-fermenting); Dias = *S. cerevisiae* var. *diastaticus* (beer spoilage)

1. WB / Alt / Koe / Ale / Dias / Lag

🡪 Beer type from BLQ and brewer experience; WB = wheat beer; Alt = Alt-beer; Koe = Kölsch; Ale; Dias = *S. cerevisiae* var. *diastaticus*; Lag = Lager

1. WB / Alt / Koe / Ale / Dias / Lag

🡪 Beer type after MALDI-TOF MS classification; WB = wheat beer; Alt = Alt-beer; Koe = Kölsch; Ale; Dias = *S. cerevisiae* var. *diastaticus*; Lag = Lager

1. YPG

🡪 Used cultivation media; Yeast Peptone Glucose

1. 18h

🡪 Aerobic growth time after pre-cultivation

1. E

🡪 Sample preparation; E = ethanol extraction with formic acid and acetonitrile

1. E_

🡪 End of labeling
